# Human Gut–Brain Interaction Chip for Dissecting the Gut-Derived LPS and Butyrate Regulation of the Blood–Brain Barrier

**DOI:** 10.3390/bios16010023

**Published:** 2025-12-29

**Authors:** Ranran Yan, Ge Gao, Yulin Deng, Jinhua Li, Yujuan Li

**Affiliations:** 1 School of Medical Technology, Beijing Institute of Technology, No. 5 Zhongguancun South Street, Haidian District, Beijing 100081, China; 2 School of Life Science, Beijing Institute of Technology, No. 5 Zhongguancun South Street, Haidian District, Beijing 100081, China

**Keywords:** gut–brain axis, three-dimensional printed, vascular decellularized extracellular matrix, lipopolysaccharide, butyrate

## Abstract

The gut–brain axis (GBA) interaction is important for human health and disease prevention. Organ chips are considered a solution for GBA research. Three-dimensional (3D) cultures and microfluidics engineered in an organ chip could improve the scientific knowledge in the GBA interactions field. In this study, a novel organ chip is developed, which achieves multicellular three-dimensional cultivation by utilizing a decellularized matrix. In addition, this paper reports the rapid prototyping process of the GBA microfluidic chip in polydimethylsiloxane (PDMS) using 3D printing interconnecting poly(ethylene/vinyl acetate) (PEVA) microchannel templates. In comparison to the static culture system of the transwell model, the intestinal epithelial barrier (IEB) and blood–brain barrier (BBB) models on our chip demonstrated superior barrier function and the efflux functionality of transporters under appropriate fluidic conditions. Additionally, it is observed that butyrate protected against BBB dysfunction induced by gut-derived lipopolysaccharide (LPS) via enhancing intestinal barrier function. These results demonstrate that this multicellular, three-dimensional cultivation integrated with a fluidic shear stress simulation chip offers a promising tool for gut–brain interaction study to predict therapy of intestinal and neurological disorders.

## 1. Introduction

The crosstalk between the gut and brain has long been recognized in health and disease. Research on the gut–brain axis (GBA) has confirmed that gut microbiota plays a key role in communication between these two distant organs [1]. Indeed, the reduction in beneficial bacteria and the accumulation of pathogenic bacteria have been implicated in a myriad of central nervous system (CNS) disorders, such as anxiety, depression, stroke, cognitive dysfunction, Alzheimer’s disease, and substance abuse [2]. The intestinal epithelial barrier (IEB) and blood–brain barrier (BBB) are present in the gut and brain and maintain strict intestinal and CNS homeostasis across GBA [3]. The malfunction of the barriers due to alterations in the composition of gut microbiota may underlie the development of neurological disorders [4]. Therefore, understanding how the gut microbiota modulates these barriers could be crucial in understanding the etiology of neurological disorders.

Research on the complex GBA has relied mainly on in vivo animal models. However, due to ethical considerations and the challenges in replicating human physiology and disease in animal models, more advanced human-based models are required for research [5]. Organ-on-a-chip (OOC) technologies have provided a way for breakthroughs and progression in the understanding of gut–brain communication [6]. Currently, only a few GBA-on-a-chip models have been reported [7,8,9,10,11,12,13]. OOCs are predominantly composed of polydimethylsiloxane (PDMS). However, the microfabrication of photoresist-based molds is complicated, expensive, and time-consuming [14]. In addition, current GBA chip models have limitations in replicating the three-dimensional growth environment of cells, and reported models rely on non-tissue-specific commercial hydrogels [7,8,9,10,11]. Second, current models based on simple co-cultures of gut epithelial and brain endothelial cells lack key required cell types, such as human intestinal microvascular endothelial cells (HUVEC), human brain vascular pericytes (HBVP), and astrocytes for barrier function, which are vital for regulating barrier function and immune regulation [7,8,9,10,11,12,13]. Furthermore, at the system integration level, few chip platforms can apply independent, physiologically relevant fluid shear stress stimuli to multiple cell types within both the gut and brain modules simultaneously. This shortcoming hinders in-depth investigation into the role of mechanical signals in bidirectional gut–brain communication [7,8,9,10,11,12,13].

This study describes a GBA chip capable of multicellular three-dimensional cultivation and fluidic shear stress simulation. Furthermore, this study presents a cost-effective and open-source methodology for the rapid prototyping of GBA microfluidic devices in PDMS, employing three-dimensional (3D) printing to fabricate interconnecting poly(ethylene/vinyl acetate) (PEVA) microchannel templates. The IEB and BBB models with superior barrier function were constructed using vascular decellularized extracellular matrix (VdECM). In addition, we confirm that butyrate (a short-chain fatty acid) protects against brain barrier dysfunction induced by gut-derived lipopolysaccharide (LPS) by enhancing intestinal barrier function.

## 2. Materials and Methods

### 2.1. Fabrication and Assembly of the GBA Chip

**Figure 1:**
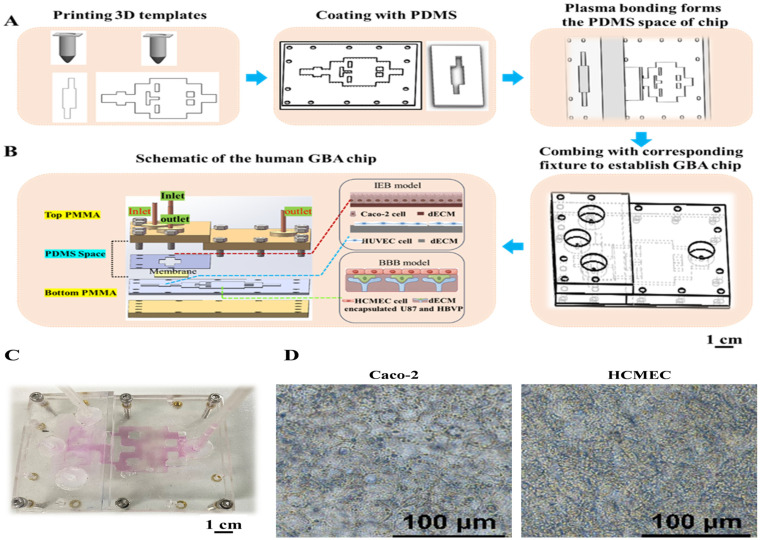
(**A**) The design of the GBA chip and the picture of the assembled GBA chip; (**B**) image of the GBA chip; (**C**) image of the assembled chip; (**D**) image of the cell morphology of Caco-2 cells and HCMECs cultured in decellularized matrix.

Three-dimensional templates fabricated by a 3D printer—a self-assembled 3D bioprinter was used to create the 3D templates. This 3D printer consists of an air pressure precision control module, a printing nozzle with an X-Z movement module, and a temperature control module. Templates for casting PDMS-based (Dow Corning, Midland, MI, USA) GBA chips were 3D-printed and consisted of both top and bottom units (Figure 1). In particular, PEVA (PolyScience, Niles, IL, USA) was heated to 120 °C for the initial printing of the GBA chip templates on glass slides. Subsequently, the templates were coated with PDMS and cured at 60 °C for 3 h. The cured PMDS parts were then assembled. A porous membrane made of polyethylene terephthalate (PET, pore size 0.45 µm, pore density 2 × 10^6^/cm^2^) was sourced from the Yichun Analysis Instrument Company (Haining, China). The PET membrane was positioned and locked in the gut cell culture area between the top and bottom units of the PDMS to allow separation between the upper and lower wells. Fixtures made of polymethylmethacrylate (PMMA; Suzhou Wenhao Microfluidic Technology Company, Suzhou, China) were prepared. The PDMS structure was then sealed using PMMA clamps and screws to create a reversibly binding matrix structure for the GBA chip.

### 2.2. Fluid Dynamics Simulation

The shear stress profiles on the GBA chip were simulated using COMSOL Multiphysics 6.1 software (COMSOL Incorporated, Stockholm, Sweden). The initial flow velocities at the channel entrance were assumed to be 2 μL/s and 35 μL/s for the IEB and BBB modules, respectively, with an injection pump (Biotaor Instrument Company, Jiaxing, China) based on the measurement of the volume flow rate.

### 2.3. Cell Preparation and Culture

Caco-2 cells obtained from the Chinese Academy of Sciences (Shanghai, China) and HUVEC from the Institute of Chinese Materia Medica (Beijing, China) were cultured in Dulbecco’s Modified Eagle Medium (DMEM) (Solarbio, Beijing, China). Human astrocytes (U87) obtained from the Beijing Institute of Technology (Beijing, China). Human brain microvascular endothelial cells (HCMEC) and HBVP sourced from iCell Bioscience (Shanghai, China) were cultured in DMEM. DMEM media was supplemented with 10% (*w*/*v*) fetal bovine serum (FBS) from Gibco (Brooklyn, NY, USA), along with 100 U/mL streptomycin, 100 U/mL benzylpenicillin, and 1% non-essential amino acid (Gibco, New York, NY, USA). All cells were cultured in a humidified 5% CO2 incubator at 37 °C.

### 2.4. Cell Seeding and Co-Culture on the Chip and Transwell

To establish the gut epithelial model on the chip, the porous membrane was coated with VdECM at 37 °C for 30 min. The VdECM obtained from porcine aortic tissue using a series of physical, chemical, and enzymatic decellularizing treatments drastically eliminated DNA content (4.92 ± 0.63%) while preserving the majority of ECM components, such as collagen (135.29 ± 6.86%), glycosaminoglycans (GAG) (75.89 ± 2.74%), and elastin (60.39 ± 7.09%) in the original tissue [15]. A total of 100 μL of Caco-2 cells (1 × 10^7^ cells/mL) were seeded onto the porous membrane of the upper culture chamber. Subsequently, the upper culture chamber was then filled with culture media, which was refreshed every other day.  From day 2 onward, the chips were connected to an injection pump fitted with micro-flow rate tubes previously sterilized by running 70% ethanol. The system comprised sterile glass reservoirs for fresh media connected to the chip inlets and the collection of spent media from the outlets. Excluding the injection pump, the setup was positioned in a cell culture incubator, and all experiments were performed at 37 °C and 5% CO_2_.

Following a 3 day dynamic culture of Caco-2 cells on the chip, the Caco-2 and HUVEC co-culture model and the HCMEC, HBVP, and U87 co-culture model were subsequently established on the GBA chip. HUVECs were digested and resuspended in the culture medium with a density of 8 × 10^5^ cells per mL, and 200 μL of cell suspension was seeded in the bottom culture chamber of the gut epithelial model. Next, a brain endothelial model on the chip was established. U87 and HBVP cells were digested and resuspended in the culture medium at a density of 5 × 10^6^ cells/mL. The two-cell suspension at a ratio of 1:1 was mixed with 1 mL of VdECM, resulting in a cell density of 1 × 10^7^ cells/mL of VdECM. Next, 500 μL of cells with the VdECM mixture were added to the right module of the chip. After 24 h of incubation at 37 °C, the HCMECs were seeded on top of the cells with the VdECM mixture for 48 h.

To establish a static culture of the gut epithelial model on the transwell, Caco-2 cells were seeded at a density of 2 × 10^5^ cells per cm_2_ on the top of a porous membrane coated with collagen and cultured in a humidified 5% CO_2_ incubator at 37 °C for 15 days. After 15 days, a transwell was inserted to seed the HUVECs. The cells were allowed to adhere to the lower surface of the transwell for approximately 5 h. Subsequently, the culture medium was added to the upper (500 μL) and the lower chambers (1.5 mL). The cells were cultured in a humidified 5% CO_2_ incubator at 37 °C. Additionally, a BBB model with three co-cultured cell lines was established in a similar manner. HBVP and U87 cells were seeded on the basolateral side of the transwell and incubated for 6 h. Subsequently, HCMECs were seeded on the apical side of the transwell.

### 2.5. Permeability Measurement

After the establishment of the brain endothelium and gut epithelial models, the apparent permeation rates (Papp) of fluorescein sodium (Sigma, St. Louis, MO, USA, Mw = 376 Da) were employed to verify the integrity of gut epithelial cells and brain endothelial cells. Briefly, 1.5 mL of Hank’s Balanced Salt Solution (HBSS) was added to the lower compartment of a chip chamber, and 0.5 mL of fluorescein sodium (1 mg/mL) was added to the upper chamber and incubated at 37 °C for 1 h in the dark. Then, 100 μL of triplicate samples from the lower chamber was collected, and its absorbance was measured at 492 nm using a BioTek Cytation3 microplate reader (BioTek Instruments, Inc., Winooski, VT, USA).

BBB permeability was evaluated following a 24 h perfusion culture by adding medium containing fluorescein sodium. After a 2 h incubation with fluorescein sodium, the VdECM matrix was washed with PBS and homogenized with trichloroacetic acid. The homogenate was centrifuged at 4 °C (12,000 rpm for 20 min), and the supernatant was immediately collected. The absorbance of the supernatant was measured at 492 nm using a BioTek Cytation3 microplate reader.

For the gut epithelial module on the transwell, the apical chamber solutions of the culture inserts were exchanged with 500 µL of HBSS containing 1 mg/mL of fluorescein sodium, while the basolateral sides of the plate inserts were filled with HBSS. The plate was then incubated at 37 °C for 30 min in the dark. Subsequently, 100 µL of triplicate samples from the basolateral chamber of each well were collected to determine the concentration of fluorescein sodium through fluorescence intensity using a BioTek Cytation3 microplate reader plate at 492 nm. The permeability test procedure for the brain endothelium model on the transwell used an accumulation assay.

### 2.6. Cell Staining and Characterization

HCMECs and Caco-2 cells were fixed, permeabilized, and blocked according to standard procedures. To visualize the tight junctions, both cell types were incubated with zonula occludens-1(ZO-1) and occludin monoclonal antibodies (Abcam, Cambridge, UK) at a 1:200 dilution, as well as glial fibrillary acidic protein (GFAP) protein monoclonal antibody (1:50) and alpha-smooth muscle actin (α-SMA) protein monoclonal antibody (1:100, Beyotime Biotechnology, Shanghai, China) in the blocking buffer for 24 h at 4 °C, and then washed with PBS. Next, the cells were incubated with goat anti-rabbit IgG (H + L) superclonal secondary antibody (1:200) for 2 h at room temperature. All nuclei were stained blue with 4,6-diamidino-2-phenylindole (DAPI, Solarbio, Beijing, China). Images were captured using an inverted laser scanning confocal microscope (Nikon Corporation, Tokyo, Japan).

### 2.7. Characterization of Efflux Functionality

The 2 μM of Rhodamine 123 (Rho123) or 20 μM of Hoechst33342 (Hoe) was used to verify the gut epithelial efflux functionality in the GBA chip or transwell. Briefly, 1.5 mL of HBSS was added to the lower compartment of a chip chamber or transwell basolateral chamber, and 0.5 mL of 2 μM Rho123 or 20 μM Hoe was added to the upper chamber in the chip or transwell apical chamber and incubated at 37 °C for 1 h in the dark. Then, 100 μL of sample from the lower chamber was collected, and the absorbance was measured using a fluorescence microscope.

The efflux functionality of the established brain endothelium model was evaluated using an accumulation assay. Rho123, or Hoe, was then added to the flow channel. After washing the VdECM matrix with PBS and homogenizing it with trichloroacetic acid, the intracellular levels of Rho123 and Hoe were quantified using a multifunctional microplate reader.

### 2.8. The Measurement of Gut-Derived LPS and Butyrate Effect on the Blood–Brain Barrier

To assess the impact of butyrate on gut epithelial and brain endothelial cell models under LPS conditions, the GBA chips were divided into the following three groups: control (CON), lipopolysaccharide (LPS), and butyrate (BUT). The GBA chips in all groups were cultured under the same conditions. The upper intestinal layer channel of the BUT group was pre-incubated with sodium butyrate (200 μM; Sigma, St. Louis, MO, USA) for 24 h. All groups, except the CON group, were treated with 10 μg/mL of LPS for an additional 24 h. To assess the protective effect of butyrate on IEB and BBB damage under LPS conditions, including permeability measurement and efflux functionality, this study also examines the expression of tight junction (TJ) and adherens junction (AJ) proteins and the efflux functionality of P-glycoprotein (P-gp) and breast cancer resistance protein (BCRP) on IEB and BBB.

### 2.9. Western Blot

Western blot analysis was performed by extracting total protein from Caco-2 and HCMEC seeded on the composite scaffolds. The total protein concentration was determined using the BCA assay, followed by denaturation in loading buffer at 100 °C for 10 min. Subsequently, total protein was separated by sodium dodecyl sulfate-polyacrylamide gel electrophoresis, transferred onto polyvinylidene difluoride membranes (Merck Millipore, Darmstadt, Germany), and blocked with 5% skim milk at room temperature for 2 h. After blocking, the membranes were incubated with primary antibodies, including β-catenin, occludin, P-gp, and BCRP (Abcam, Cambridge, UK). On the second day, horseradish peroxidase conjugated with a secondary antibody was used to incubate the membranes for 2 h at room temperature. After incubation, the protein bands were visualized using an enhanced chemiluminescence (ECL) kit (Millipore), and the protein strip intensity was analyzed using the ChemiDocTM Touch Imaging System (Bio-Rad, Hercules, CA, USA).

### 2.10. Enzyme-Linked Immunosorbent Assay (ELISA)

IL-8 levels in the culture supernatant were measured with a commercial ELISA kit (YM-S0088, Shanghai Yuanmu Biotechnology Company, Shanghai, China) according to the manufacturer’s protocol. Briefly, samples and standards were added to the assay plate. Following incubation and washes, a biotinylated detection antibody and streptavidin-HRP were added. After adding the TMB substrate and stop solution, the absorbance was detected at 450 nm with the BioTek Cytation3 microplate reader. Sample concentrations were calculated based on the standard curve.

### 2.11. Statistical Analysis

Statistical analysis was performed using SPSS 20.0 software (IBM, New York, NY, USA), and the results were expressed as mean ± SD. The difference between the two groups was determined by a one-way analysis of variance, and a *p*-value of <0.05 was considered to indicate statistical significance.

## 3. Results

### 3.1. Multicellular Three-Dimensional Cultivation Integrated with Fluidic Shear Stress Simulation Gut–Brain Axis Chip Fabrication

This study describes a novel approach that integrates a multicellular 3D culture system with fluidic shear stress simulation on a GBA chip. Firstly, this paper reports a low-cost and open-source process for the rapid prototyping of a GBA microfluidic chip in PDMS using 3D printing interconnecting microchannel scaffolds. Furthermore, the present GBA chip achieved multicellular three-dimensional cultivation by utilizing a decellularized matrix. Figure 1A shows a schematic of the process for preparing the GBA microfluidic chip using this method. A photograph of a finalized organ chip is shown in Figure 1C. The results demonstrate that the 3D-printed PEVA material template could be securely bonded to glass, allowing the construction of versatile templates that meet diverse application requirements. Furthermore, templates prepared with PEVA do not require complex thermal or chemical treatments to ensure the optimal release of PDMS modules. As shown in Figure 1A, the 3D-printed PEVA template enables the straightforward replication of units for cultivating intestinal cells and designated areas for the BBB model with PDMS. This configuration can be combined with the corresponding fixture to establish an upper and lower model, which provides a solution for performing essential operations, such as sealed perfusion culture, in future 3D-printed organizational structures. In summary, we described a cost-effective and simple method for fabricating PDMS-based microfluidic devices by combining 3D-printed PEVA templates with replica molding technology.

**Figure 2:**
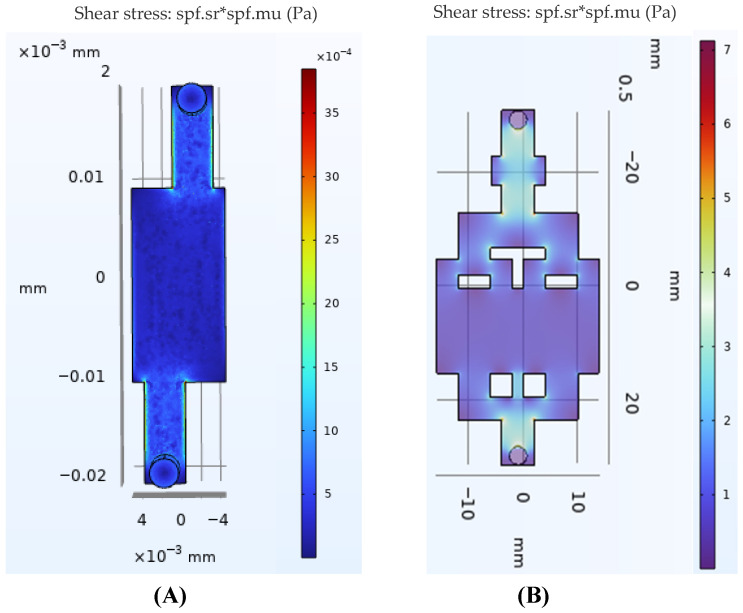
COMSOL Multiphysics simulation of fluidic shear stress inside the GBA chip. Simulation for (**A**) IEB model and (**B**) HUVEC and BBB model. Note: Shear stress is computed as the product spf.sr * spf.mu (where * is the multiplication operator).

To demonstrate the utility of the proposed microfabrication technology, a microfluidic GBA chip was constructed using the proposed method. GBA chips were designed with upper and bottom 3D-printed templates to incorporate a porous membrane within the gut model of the GBA chip (Figure 1B). The upper template included a couple of inlets and outlets and a Caco-2 cell culture area, while the bottom template contained a couple of inlets and outlets, an HUVEC culture area, an interactive channel, and a BBB module. The intermediate interaction channel was designed to simulate the shear stress on umbilical vein endothelial cells and brain microvascular endothelial cells using a shared perfusion inlet. In this study, the constructed BBB model involves co-embedding astrocytes and pericytes within VdECM, with the expression of their specific marker proteins confirmed by immunofluorescence (IF) staining (Figure S3). Brain microvascular endothelial cells were seeded onto the surface of the VdECM to establish a three-dimensional co-culture model incorporating all three cell types. The cell morphology of gut–brain-related cells cultured in the decellularized matrix is shown in Figure 1D. The spatial distribution of the shear stress levels within the chip was predicted using COMSOL Multiphysics simulation. Biomechanical shear stress plays a vital role in the function recapitulation in vitro intestinal and BBB models. COMSOL Multiphysics was used to perform computational fluid dynamics (CFD) simulations to replicate physiological shear stress in our GBA chip. For the intestinal barrier model, the simulation results demonstrated that a perfusion flow rate of 2 µL/min at the inlet generated the anticipated values of shear stress in the IEB module, varying 2.2 × 10^−5^ dyne/cm^2^ (Figure 2A). This closely matched the reported shear stress between 0.4 × 10^−5^ and 1.33 × 10^−4^ dyn/cm^2^ for enhancing the barrier function of Caco-2 cells [7]. Shear stress levels within the brain microvasculature ranged from 4 to 20 dyne/cm^2^ [16]. The predictive results of the BBB module indicated that at an inlet flow rate of 35 µL/min, the applied shear stress on brain microvascular endothelial cells reached 6 dyn/cm^2^ (Figure 2B), thus enabling the in vitro simulation of physiological shear stress. In the present work, we focus on the establishment of a three-dimensional multicellular culture IEB and BBB model suitable for dynamic culture in a developed GBA chip.

### 3.2. Intestinal Epithelial Model on the Chip Recapitulates Superior Barrier Function

**Figure 3:**
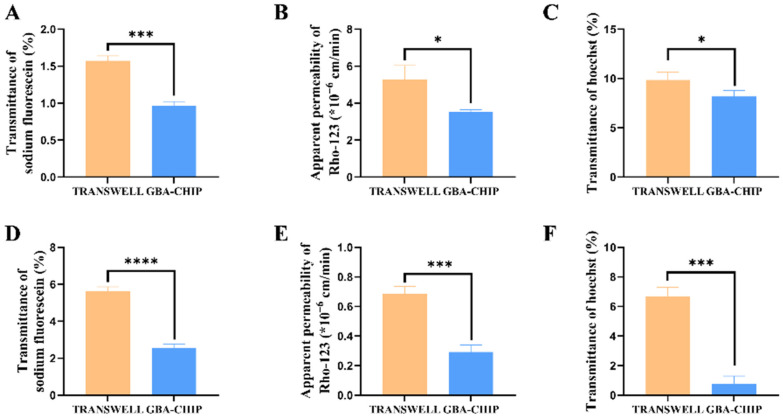
Assessment of intestinal epithelial model barrier function on GBA chip. (**A**) Graph on permeability measurement. Comparison of permeability value on transwell cultured cells. (**B**) Graph of transporter P-gp functionality on both the chip and the transwell. (**C**) Graph on transporter BCRP functionality on both the chip and transwell. Assessment of the brain endothelium model barrier function model on the GBA chip. (**D**) Graph on permeability measurement. Comparison of permeability value on transwell cultured cells. (**E**) Graph on transporter P-gp functionality on both the chip and the transwell. (**F**) Graph on transporter BCRP functionality on both the chip and the transwell. The values represent mean ± SD. Note: * *p* < 0.05, *** *p* < 0.006, **** *p* < 0.001, compared with the transwell model group.

**Figure 4:**
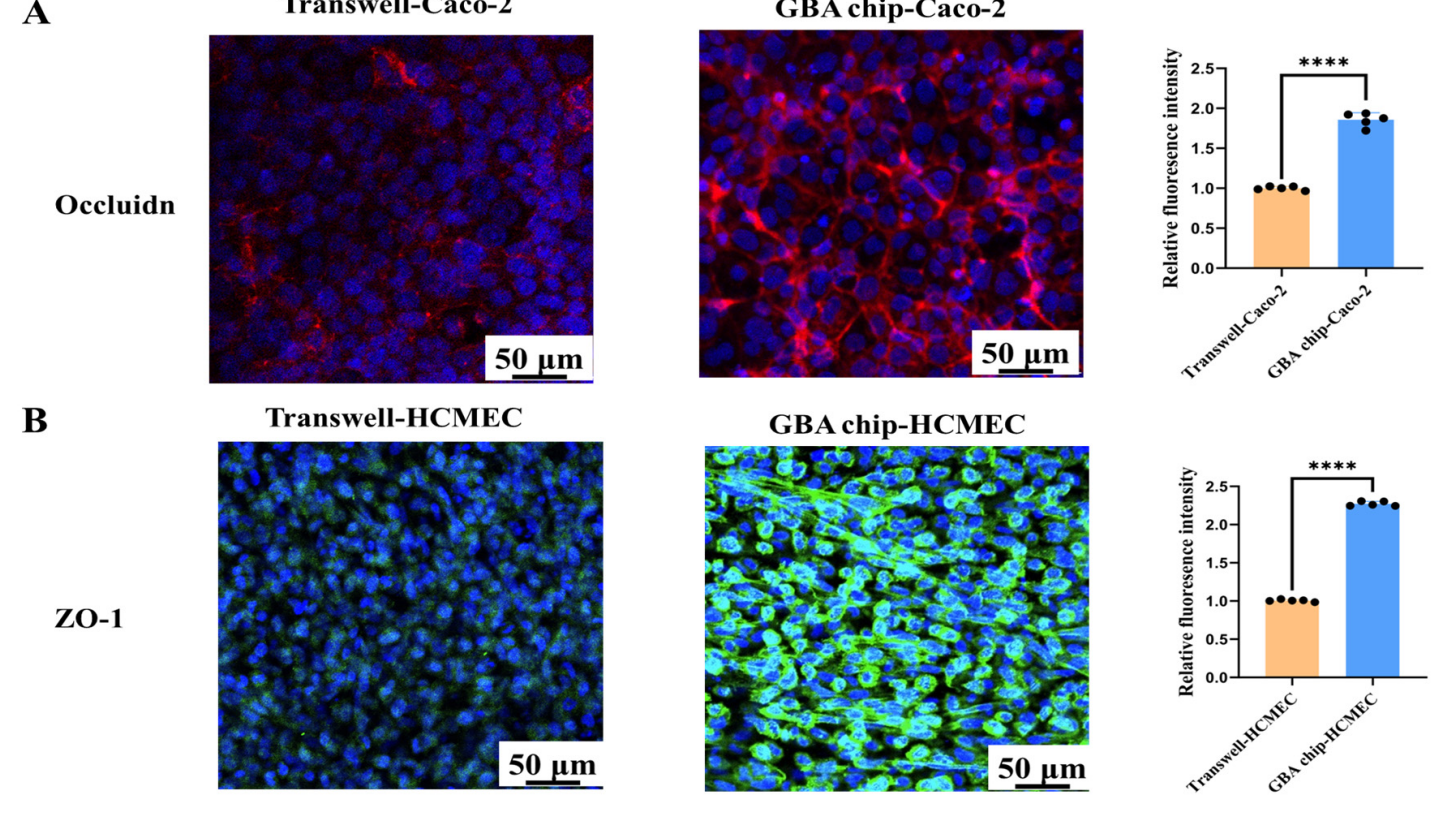
Assessment of intestinal epithelial model barrier function and brain endothelium model barrier function on the GBA chip. (**A**) Top views of the Caco-2 cell layer of tight junction occludin in the transwell and in the GBA chip. (**B**) Top views of the HCMEC layer of tight junction ZO-1 in the transwell and in the GBA chip. The values represent mean ± SD. Note: **** *p* < 0.001, compared with the transwell model group.

The transwell model was static cultured for 21 days, and the IEB model in the GBA chip was exposed to fluidic flow for 5 days. Permeability was evaluated using fluorescence spectroscopy. Fluorescein was absorbed approximately 5-fold less when Caco-2 cells were co-cultured with HUVEC in the chip compared to a co-culture in a transwell (Figure 3A). This result suggested that the mechanical stimulus and VdECM matrix contributed to the reduction in permeability in this chip. In addition to passive paracellular absorption in the influx direction, another important characteristic of IEB function is efflux transporter activity. Figure 3B shows the efflux transport of Rho123 (a P-pg substrate) across the Caco-2 cell layer in both the GBA chip and transwell models. The efflux ratio was 10-fold higher in the GBA chip than in the static transwell culture. In addition, we evaluated the function of the efflux transporter BCRP in Caco-2 monolayers cultured in the microfluidic system. The efflux ratio of Hoe in the GBA chip was 0.82-fold higher than that in the static transwell culture (Figure 3C). The integration of fluidic stimulation and 3D culture within the chip increased the efflux transport activity of P-gp and BCRP. Additionally, the research also found that the application of VdECM significantly increased the expression of TJ proteins in Caco-2 cells. IF results further demonstrate that relative to statically cultured Caco-2 cells, Caco-2 cells cultured under shear stress simulation with VdECM expressed increased levels of occludin than the transwell model (Figure 4A). These findings demonstrated that the application of fluidic shear stress and the use of a decellularized matrix enhanced the barrier function of intestinal cells.

### 3.3. Brain Endothelium Model on the Chip Recapitulates Superior Barrier Function

This study also assesses brain barrier functionality on two platforms, including three-dimensional cultivation with constant-rate flow on the chip and static culture on the transwell. Compared to the transwell model, the permeability of fluorescein was significantly lower in the chip-based model. The transmittance ratios were measured as 5.69% for the transwell and 2.56% for the chip. (Figure 3D). IF results also demonstrated that the expression of the intercellular junction protein ZO-1 in HCMEC was significantly higher on the GBA chip than on the transwell model (Figure 4B). Furthermore, the barrier function of the BBB model was evaluated by assessing transporter functionality in both chip-based and transwell systems. The accumulation of Rho123 and Hoe in the chip was significantly lower than that in the transwell assay (Figure 3E,F). Rho123 accumulation decreased from 0.68% to 0.30%, and Hoe decreased from 6.68% to 0.78%. Therefore, the chip platform enhanced the efflux functionality of the ATP-binding cassette (ABC) transporters compared to the transwell. All the data indicated that the three-dimensional cultivation with constant-rate flowing on the chip could significantly improve the barrier function of the brain endothelium model.

### 3.4. Butyrate Protected Against BBB Dysfunction Induced by LPS Through Enhancing IEB Function

**Figure 5:**
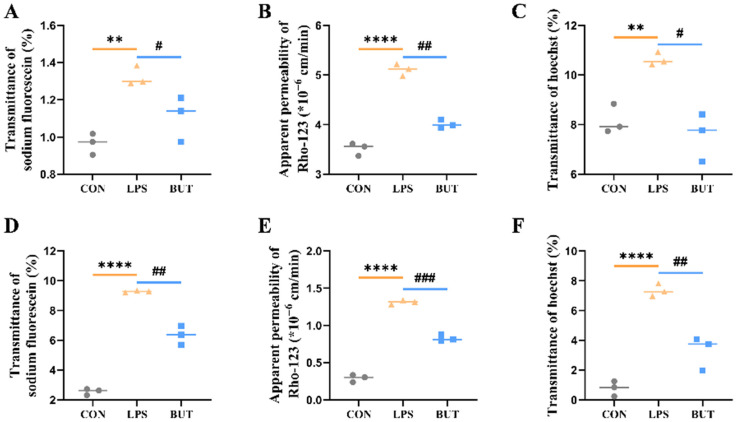
Protection in the intestinal epithelial model barrier function of Caco-2 cells by butyrate in the GBA chip under the LPS effect. (**A**) Graph on permeability measurement. (**B**) Graph on transporter P-gp functionality on a chip. (**C**) Graph on transporter BCRP functionality on both the chip and the transwell. Protection in the brain endothelium model of HCMEC by butyrate in the GBA chip under LPS effect. (**D**) Graph on permeability measurement. (**E**) Graph on transporter P-gp functionality on a chip. (**F**) Graph on transporter BCRP functionality on both the chip and the transwell. The values represent mean ± SD. Note: ** *p* < 0.005, **** *p* < 0.001, compared with normal control. # *p* < 0.05, ## *p* < 0.005, ### *p* < 0.001, compared with the LPS group.

This study employs a GBA chip to investigate the in vitro regulation of the BBB by gut-derived LPS and butyrate. The BBB and IEB functions were evaluated by adding LPS and butyrate to the top layer of the gut chip. Permeability test results revealed a significant decrease in the butyrate treatment group compared to the LPS group in the IEB model (Figure 5A).

**Figure 6:**
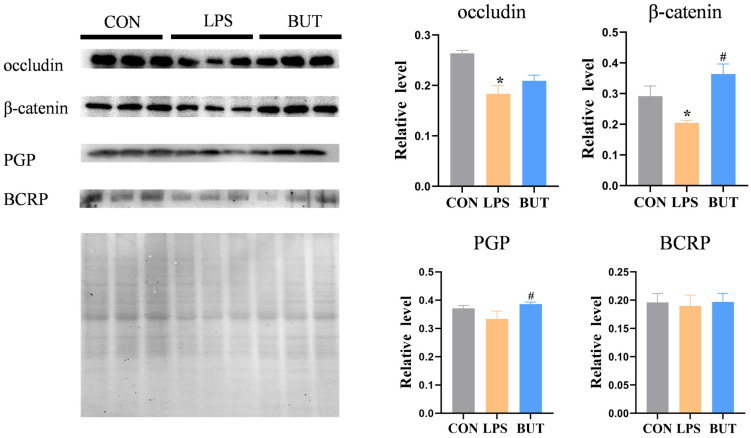
Western blot analysis of occludin, β-catenin, P-gp, and BCRP expression under the intestinal epithelial model of the GBA chip. The values represent mean ± SD. Note: * *p* < 0.05, compared with the normal control group. # *p* < 0.05, compared with the LPS group.

**Figure 7:**
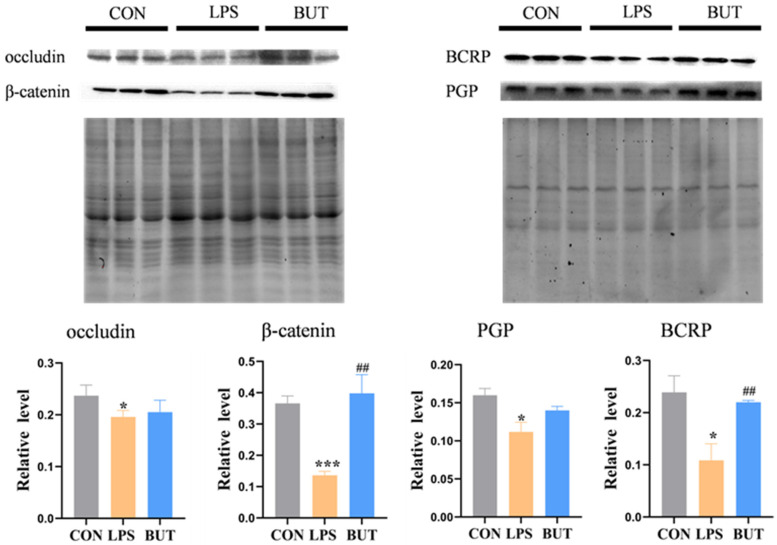
Western-blot analysis in occludin, β-catenin, P-gp and BCRP expression under brain endothelium model of GBA chip. The values represent mean ± SD. Note: * *p* < 0.05, *** *p*< 0.001, compared with normal control group. ## *p* < 0.005, compared with LPS group.

The permeability data of the LPS- and butyrate-treated groups were 1.32% and 1.10%, respectively. TJs and AJs play important roles in maintaining the integrity of the intestinal barrier. Consistent with the results for permeability, the expression of occludin and β-catenin significantly increased by 14% and 78% in the butyrate-treated groups, respectively, compared to that in the LPS group (Figure 6). In addition, the results indicated that butyrate significantly decreased the permeability of fluorescein in the BBB model (Figure 5D). Furthermore, the protective effect of butyrate on BBB dysfunction was evaluated by examining the expression of TJ proteins. Western blotting showed that butyrate treatment dramatically upregulated the expression of occludin and β-catenin in the butyrate-treated group compared to that in the LPS group (Figure 7).

To further elucidate the protection by butyrate, we examined the effect of butyrate on the transport of Rho123 (a P-pg substrate) and Hoe (a BCRP substrate) in the gut model, the results of which are shown in Figure 5B,C. The results indicated that butyrate significantly increases the efflux rate of Rho123 and Hoe compared with the LPS group. In addition, butyrate significantly reduced the intracellular accumulation of Rho123 and Hoe in the BBB model (Figure 5E,F). P-gp and BCRP levels were downregulated by treatment with LPS for 24 h, as determined by Western blotting (Figure 6). Butyrate (200 μM) significantly enhanced P-gp and BCRP expression in both caco-2 of the gut model (Figure 6) and HCMEC of the BBB model (Figure 7).

**Figure 8:**
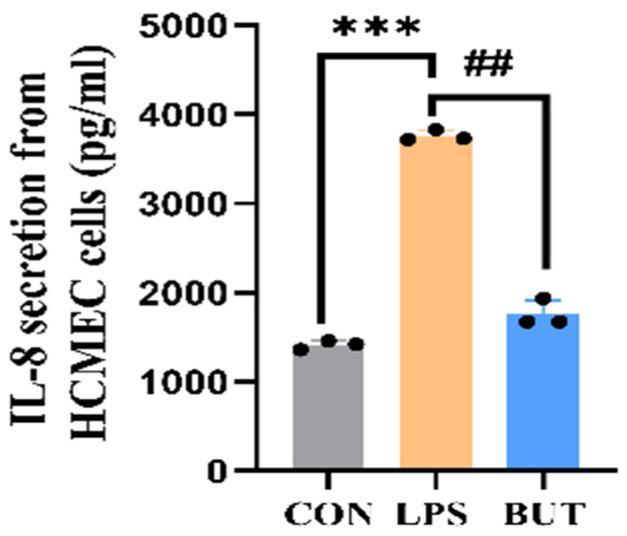
LPS-induced changes in cytokine secretion of HCMECs in the GBA chip model. The values represent mean ± SD. Note: *** *p* < 0.001, compared with the normal control group. ## *p* < 0.005, compared with the LPS group.

Using the gut–brain axis chip, we confirmed the potential impact of gut-derived endotoxin stimulation on cytokine secretion in the brain. The present results reveal that exposure to 10 μg/mL LPS resulted in intestinal barrier damage, which increased the secretion of IL-8 from HCMEC (Figure 8). In the gut–brain axis chip, we confirmed the potential impact of gut-derived endotoxin stimulation on cytokine secretion in the brain. The present results reveal that exposure to 10 μg/mL LPS resulted in intestinal barrier damage, which significantly increased the secretion of IL-8 from HCMEC (Figure 8). In the present study, we investigate the anti-inflammatory effect of butyrate on the production of IL-8 in a BBB model of a GBA chip treated with LPS. These results show that butyrate inhibited LPS-induced IL-8 production in the GBA chip.

## 4. Discussion

This study introduces a low-cost and open-source process for the rapid fabrication of a PDMS-based GBA microfluidic chip with 3D-printed scaffolds, enabling 3D multicellular culture using decellularized matrices. PDMS-based devices are commonly employed in microfluidic applications because of their biocompatibility, optical clarity, and gas permeability. Traditionally, PDMS devices are cast using molds fabricated by photolithography of SU-8 on silicon micromachine wafers [17]. This technique requires sufficient equipment, such as spin coaters for SU-8 substrates. To simplify template fabrication, we demonstrated the ability to construct a GBA chip with a PDMS-based unit cast on 3D-printed PEVA templates. In recent years, wax-based 3D-printed molds have been limited by the time-consuming thermal and chemical treatments of the 3D-printed master and the limited number of molding cycles. This study shows that 3D-printed PEVA templates can be securely bonded to glass for multi-use, eliminating the need for complex treatments to facilitate PDMS demolding. In summary, we described a cost-effective and simple method for fabricating PDMS-based microfluidic devices by combining 3D printing PEVA templates with replica molding technology.

To demonstrate the utility of the proposed microfabrication technology, we employed microfluidic devices constructed using this technology to produce a GBA chip. The integration of a porous substrate to separate two parallel microchannels has been demonstrated to be effective in analyzing critical physiological parameters, including tissue barrier function, transcellular transport, absorption, and secretion, in various OOAC applications for the lung, intestine, and BBB [18,19,20]. Consequently, we designed upper and bottom 3D-printed templates to incorporate a porous membrane within the gut model of the GBA chip. The currently developed GBA chips have limitations in replicating the three-dimensional growth environment of cells and lack the required cell types, such as HBVP and astrocytes, for barrier function [7,8,9,10,11,12,13].

Despite their simplicity compared to in vivo situations, 3D models have significantly contributed to the recapitulation of tissue properties, cell–cell contacts, and cell–extracellular matrix (ECM) interactions. VdECMs are excellent candidates for mimicking the ECM of soft tissues and show great potential for the development of 3D brain-like tissue models. Several studies have indicated that soft hydrogels support the differentiation and survival of astrocytes and HBVP [21,22]. For the current GBA chip, HCMECs have been cultured alone in devices, and the role of glial cells and HBVP has fallen into the background. Now it has clearly emerged that the advanced modeling of the BBB depends on the co-culture of different neural cell populations and the investigations of their interactions [23]. For instance, astrocytes play key roles in the formation of the blood–brain barrier, such as increasing the expression of TJ proteins [24]. In the present work, we focused on setting up a three-dimensional multicellular culture IEB and BBB model suitable for dynamic culture in a developed GBA chip.

The PEVA templates used in this study were fabricated via Fused Deposition Modeling (FDM). The inherent layer-by-layer deposition characteristic of this process introduces micrometer-scale roughness on the structural surfaces [25]. This study employs COMSOL simulations based on the ideal wall assumption to reveal the trends and approximate magnitudes of shear stress across different modules. However, it should be noted that the actual surface roughness disturbs the near-wall flow field, potentially inducing local vortices and variations in flow velocity gradients, which may consequently cause the local shear stress in certain regions to deviate from the model predictions [26]. Theoretically, such flow field disturbances could further influence the morphology, adhesion behavior, and related functional responses of cells. It is important to emphasize that all devices used for the experimental groups in this study were fabricated under completely identical printing processes and parameters. Therefore, the flow field variations introduced by surface roughness are relatively consistent across groups. Furthermore, the existing literature indicates that the physiological shear stress ranges for simulating the in vitro environments of the intestinal epithelium and cerebral endothelium are approximately 0.4 × 10^−5^–13.3 dyn/cm^2^ and 4–20 dyn/cm^2^, respectively [7,16]. The shear stress values derived from our simulation, based on the smooth-wall assumption, fall entirely within this physiological range (2.2 × 10^−5^ dyn/cm^2^ of IEB and 6 dyn/cm^2^ of BBB). Moreover, following exposure to the simulated shear stress, the chip model exhibited upregulation of tight junction and transporter protein expression, as well as low barrier permeability, as detected by immunofluorescence and Western blotting. Together, these findings indicate that, despite the limitations posed by surface roughness, the data provided by the current COMSOL simulations remain acceptable.

This study further evaluates whether the 3D VdECM-based IEB and BBB models adequately recapitulate the key biochemical parameters of barrier function in vitro. To achieve this, we tested the permeability, efflux transport activity of P-gp and BCRP, and expression of tight junction proteins (occludin, β-catenin, and ZO-1) using a conventional static transwell model. In many intestinal and CNS systemic pathologies, there is an elevation in intestinal or brain endothelial permeability, which corresponds to a reduction in the barrier function. Fluorescein was absorbed more when Caco-2 cells were co-cultured with HUVECs in a chip compared to a co-culture in a transwell. Brain endothelial permeability significantly decreased under dynamic flow conditions in the GBA chip. Other research groups have characterized the integrity and differentiation of Caco-2 cell layers in microfluidic chips [27,28]. The present chip also demonstrated a trend towards superior barrier function compared to the transwell models. TJs play an important role in the intestinal and brain endothelial barrier functions. TJs in intestinal epithelial cells, comprising proteins such as occludin, regulate the paracellular permeability of water, ions, and macromolecules in adjacent cells. IF staining results show that relative to statically cultured Caco-2 cells, Caco-2 cells cultured under shear stress simulation with VdECMs express higher levels of occludin than those cultured in the transwell model. The BBB is composed of cerebral microvessel endothelial cells with highly impermeable TJs, also known as ZO-1, forming a selective barrier between neighboring compartments. IF results also demonstrate that the expression of the intercellular junction protein ZO-1 in HCMEC was significantly higher on the GBA chip than on the transwell model. The shear stress induced by the fluidic flow has the effect of the mechano-transduction in several epithelial molecular pathways by activating membrane-bound receptors, leading to upregulation of the expression of the tight junction proteins (e.g., occludin). Alcaide et al. discovered that incorporating laminin and hyaluronan into hydrogels enhanced the expression of the tight junction protein ZO-1 in endothelial cells [29]. Kumar et al. successfully developed gelatin-based bio-composite films that emulate the extracellular matrix, facilitating the differentiation of Calu-3 and mesenchymal stem cells into epithelial cells characterized by tight junction protein expression and barrier formation [30].

In addition to having paracellular absorption in the influx direction, another important characteristic of BBB function is efflux transporter activity. P-gp and BCRP transporters are the best-characterized transporters in the ABC superfamily and play a pivotal role in the barrier of the gut and brain tissues owing to their efflux mechanism. P-gp and BCRP transporters change the expression and function of IEB and BBB, which are of great importance in drug absorption and distribution and barrier integrity maintenance. Next, we evaluated the functionality of the transporters P-gp and BCRP at the IEB and BBB within both chip-based and transwell systems. The cao-2 cells and HCMECs also exhibited significantly greater efflux transporter activity, and withmore closely mimicked the in vivo responses than the results obtained with cells maintained under conventional culture conditions. The Vriend group discovered that human proximal tubule epithelial cells (PTECs) cultured under flow conditions with a 3D tubular structure showed increased expression of the organic cation transporter (OCT2), making it more suitable for high-throughput drug screening and further improving the predictive accuracy of the model [31]. The use of kidney-on-a-chip platforms has been shown to enhance the basal characteristics of PTECs due to the inclusion of physiological fluid shear stress and cell–extracellular matrix (ECM) interactions, which in turn leads to increased P-gp efflux activity [32]. In conclusion, GBA chips exhibit superior IEB and BBB function compared to transwell models, which is likely due to the inclusion of physiological fluid shear stress and cell–extracellular matrix (ECM) interactions. These conditions have been shown to enhance barrier properties and the expression of tight junction proteins, such as ZO-1, as well as the activity of efflux transporters, such as P-gp, thereby improving the predictive accuracy of the model for applications.

In this study, we employ a gut–brain chip to investigate the in vitro regulation of the BBB by gut-derived LPS and butyrate. LPS, the main endotoxin produced by symbiotic Gram-negative bacteria in the intestinal tract, can enter the circulatory system and lead to CNS inflammation [33]. Currently, LPS-induced models are extensively employed to investigate the impact of enterogenic inflammation on neurological disorders. Zhao et al. demonstrated that fecal microbiota transplantation protects against rotenone-induced Parkinson’s disease in mice by suppressing inflammation mediated by the lipopolysaccharide-TLR4 signaling pathway through the microbiota–gut–brain axis [34]. Yu et al. reported that gut-derived bacterial LPS attenuates incubation of methamphetamine craving by modulating microglia [35]. Butyrate, a short-chain fatty acid (SCFA) produced by symbiotic bacteria in the gastrointestinal tract through the fermentation of dietary fibers, has been demonstrated to possess multiple benefits, including enhancing the gut barrier, reshaping the gut microenvironment, and repressing inflammatory progression [36]. The present study finds that butyrate (200μM) protects against LPS-induced BBB disruption by decreasing permeability, upregulating TJ protein expression, repairing P-gp and BCRP efflux transporter activity, and enhancing P-gp and BCRP expression in caco-2 of the gut model. In the GBA chip BBB model, treatment with butyrate impaired compromised BBB, which exhibited reduced permeability, enhanced efflux transport activity, and elevated expression of tight junction proteins (occludin and β-catenin) and transporters such as P-gp and BCRP.

This study finds that butyrate significantly upregulates the expression of junction proteins and transporter proteins in an LPS-induced BBB injury model. This protective effect may stem from the dual targeting of butyrate on G protein-coupled receptors (GPCRs) and histone deacetylases (HDACs), constituting a synergistic network that ranges from rapid anti-inflammatory action to long-term genetic reprogramming. First, as an endogenous ligand for GPR41/43, butyrate can modulate the activity of GPCRs and suppress the activation of key pro-inflammatory signaling pathways. This, in turn, alleviates the LPS-induced inflammatory cascade and establishes a favorable microenvironment for barrier repair. Research further indicates that in the BBB, butyrate-sensitive receptors (e.g., GPR109A) can directly promote the upregulation of tight junction proteins, including occludin, claudin-1, and ZO-1, thereby consolidating barrier integrity [37]. Additionally, as a histone deacetylase inhibitor, butyrate enters the nucleus, induces histone hyperacetylation, promotes the transcription of genes related to tight junction proteins and transporter proteins at the genetic level, and ultimately enhances their protein expression and functional activity [38].

Current research has found that the pro-inflammatory cytokine IL-8 is involved in GBA interactions, and the BBB is an important source of IL-8. First, we measured IL-8 levels after adding LPS to the intestinal chip to confirm the potential impact of gut-derived endotoxin stimulation on cytokine secretion in the brain. The present results reveal that exposure to 10 μg/mL LPS resulted in intestinal barrier damage, which in turn significantly increased the secretion of IL-8 from HCMECs, indicating that this chip can be used to explore the impact of intestinal injury on the BBB. Next, we investigated the anti-inflammatory effect of butyrate on the production of IL-8 in the BBB model of the GBA chip treated with LPS. These results showed that butyrate inhibited LPS-induced IL-8 production in the GBA chip. A recent study found that butyrate could modulate the activity of GPRs and inflammation-related pathways such as NF-κB and JAK/STAT, thereby reducing the release of pro-inflammatory cytokines, inhibiting intestinal inflammatory responses, and maintaining intestinal immune homeostasis [39,40]. In the present study, we discovered that butyrate might exert its anti-inflammatory effect on endothelial cells of the BBB through the transmission of the gut–brain axis, leading to the suppression of inflammatory cytokine release. This finding suggests that butyrate, a short-chain fatty acid produced by the gut microbiota, could play a crucial role in modulating neuroinflammation and emphasizes the importance of the gut–microbiota–brain axis in maintaining immune homeostasis.

Although a number of studies have demonstrated the in vivo effects of microbe-derived SCFA on neurogenesis or neurodegenerative diseases. Impaired BBB function is a critical feature involved in the development of neurodegenerative diseases. However, it is unclear whether butyrate can further protect BBB function in an in vitro IEB model. The results obtained from this in vitro study provide a foundational understanding that complements the findings of previous in vivo experiments [41], aiming to confirm the protective effect of butyrate on LPS-induced BBB dysfunction by enhancing intestinal barrier function in the GBA chip.

## 5. Conclusions

To the best of our knowledge, only a few GBA-on-chip models have been reported. However, the microfabrication of photoresist-based GBA chips is complicated, expensive, and time-consuming. In addition, the currently developed GBA chips have limitations in replicating the three-dimensional growth environment of cells and lack the required cell types, such as HBVP and astrocytes, for barrier function. Here, we present a prototypic GBA-on-a-chip with co-cultured IEB and BBB cells. IEB and BBB models with good barrier functions were constructed using decellularized extracellular matrix and fluidic shear stress simulations. Furthermore, we introduced a low-cost and straightforward method for fabricating PDMS-based GBA microfluidic devices by combining 3D-printed templates with replica molding technology. Testing the barrier function of the IEB and BBB models on the chip demonstrated superior function compared to that of the transwell model. Additionally, butyrate protected against LPS-induced BBB dysfunction by enhancing intestinal barrier function. Butyrate protected against IEB disruption by lowering permeability, enhancing efflux transport function, and upregulating the expression of tight and adherens junction proteins and efflux transport proteins. The obtained results indicated that our VdECM-based IEB and BBB models recapitulated the key biochemical parameters of barrier function in vitro. Furthermore, this chip facilitates a thorough analysis of the transport of gut-derived substances across the intestinal barrier and BBB, as well as their impact on the BBB. The protective effect of butyrate on the BBB highlights the significance of gut-derived metabolites in brain health and disease. We believe that our innovative gut–brain axis chip has the potential to serve as a valuable in vitro platform for exploring gut–brain interactions.

## Data Availability

The original contributions presented in this study are included in the article/Supplementary Materials. Further inquiries can be directed to the corresponding authors.

## Supplementary Materials

The following supporting information can be downloaded at: https://www.mdpi.com/article/10.3390/bios16010023/s1, Figure S1: Effects of butyrate on protein expression of occludin, β-catenin, P-gp, and BCRP in Caco-2 as determined by Western blot.; Figure S2: Effects of butyrate on protein expression of occludin, β-catenin, P-gp, and BCRP in HCMEC as determined by Western blot.; Figure S3: Immunofluorescence images for GFAP (marker for astrocytes) and α-SMA (marker for pericytes).

## Abbreviations

The following abbreviations are used in this manuscript:

GBA	Gut–Brain Axis
3D	Three-dimensional
PDMS	polydimethylsiloxane
PEVA	poly (ethylene/vinyl acetate)
IEB	Intestinal epithelial barrier
BBB	Blood–Brain Barrier
LPS	lipopolysaccharide
CNS	Central nervous system
OOC	Organ-on-a-chip
HBVP	Human brain vascular pericytes
VdECM	Vascular decellularized extracellular matrix
TJs	Tight junctions
ABC	ATP-binding cassette
AJs	Adherens junctions
BCRP	Breast cancer resistance protein
HUVECs	Human umbilical vein endothelial cells
DMEM	Dulbecco’s Modified Eagle’s Medium
HCMECs	Human brain microvascular endothelial cells
