# Supplementary material for: Human Gut–Brain Interaction Chip for Dissecting the Gut-Derived LPS and Butyrate Regulation of the Blood–Brain Barrier
Source: Biosensors (Basel). 2025 Dec 29;16(1):23. doi: 10.3390/bios16010023 (PMC12839142; doi:10.3390/bios16010023)
Supplement: Supplementary file 1 [file biosensors-16-00023-s001.zip › Supplementary Figure S1.pdf]

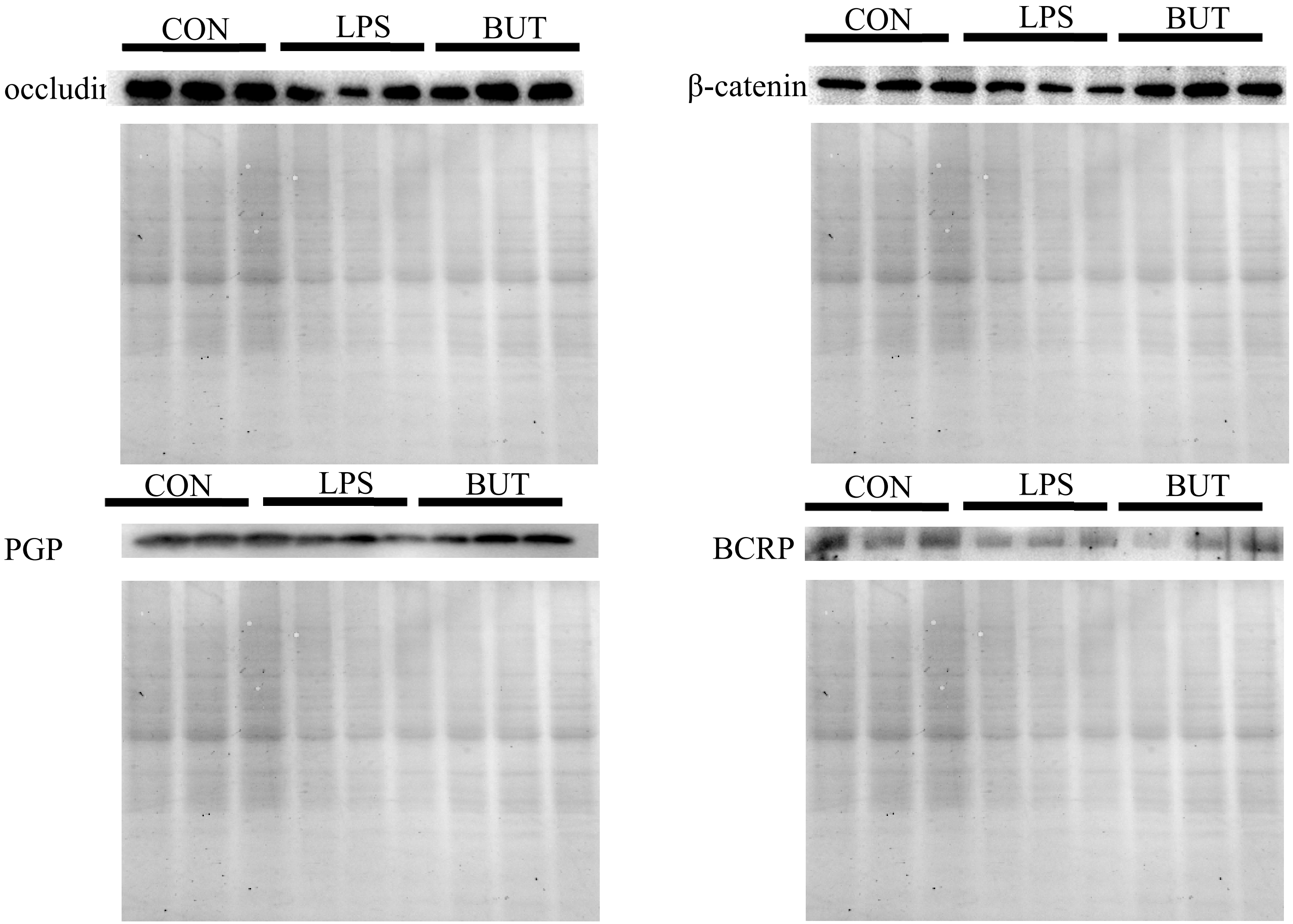
CON
LPS
BUT
occludi
CON
LPS
BUT
β-catenin
CON
LPS
BUT
PGP
CON
LPS
BUT
BCRP

Supplementary Figure S1. Effects of butyrate on protein expression of occludin, β-catenin, P-gp, BCRP in Caco-2 as determined by Western blot.
