# Supplementary material for: Human Gut–Brain Interaction Chip for Dissecting the Gut-Derived LPS and Butyrate Regulation of the Blood–Brain Barrier
Source: Biosensors (Basel). 2025 Dec 29;16(1):23. doi: 10.3390/bios16010023 (PMC12839142; doi:10.3390/bios16010023)
Supplement: Supplementary file 1 [file biosensors-16-00023-s001.zip › Supplementary Figure S2.pdf]

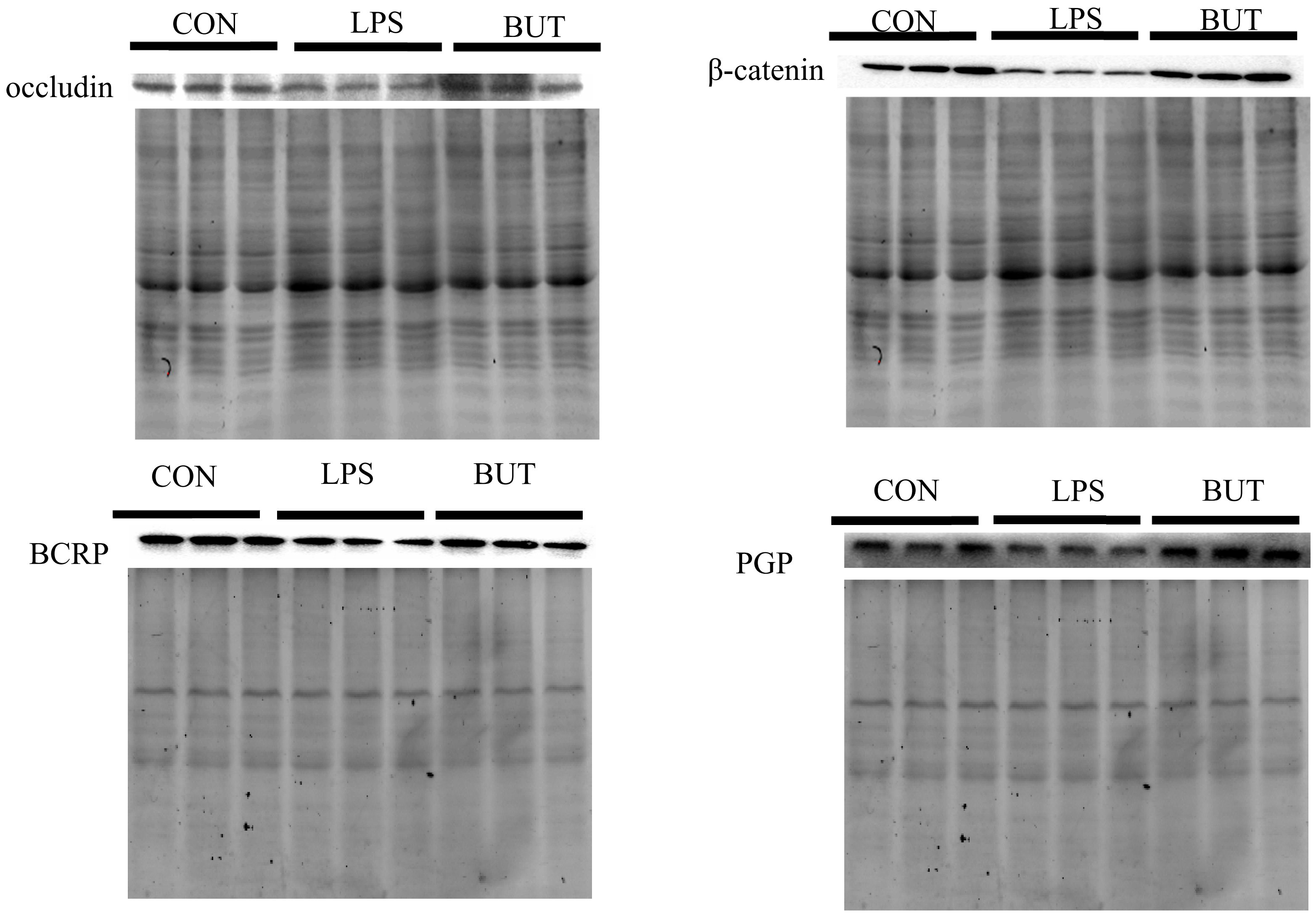
CON
LPS
BUT
occludin
CON
LPS
BUT
β-catenin
CON
LPS
BUT
BCRP
CON
LPS
BUT
PGP

Supplementary Figure S2. Effects of butyrate on protein expression of occludin, β-catenin, P-gp, BCRP in HCMEC as determined by Western blot
