# Supplementary figures and images for: Human Gut–Brain Interaction Chip for Dissecting the Gut-Derived LPS and Butyrate Regulation of the Blood–Brain Barrier
Source: Biosensors (Basel). 2025 Dec 29;16(1):23. doi: 10.3390/bios16010023 (PMC12839142; doi:10.3390/bios16010023)

# Western blot bands and original gels in cell

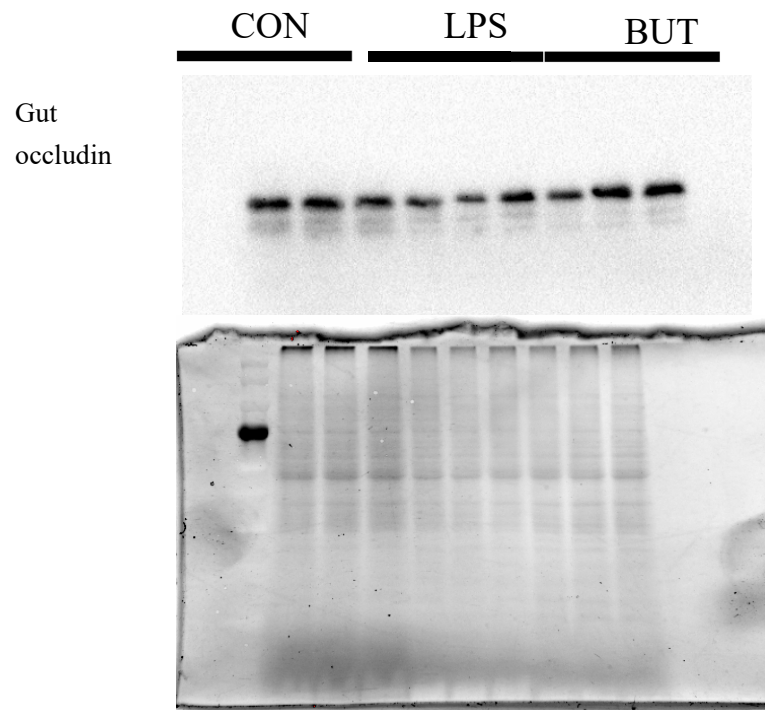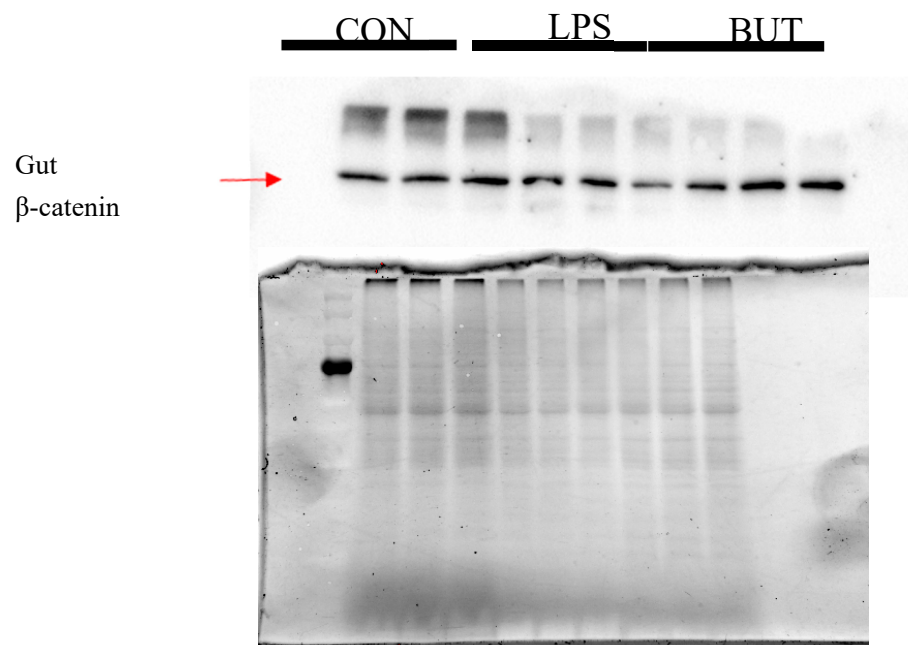

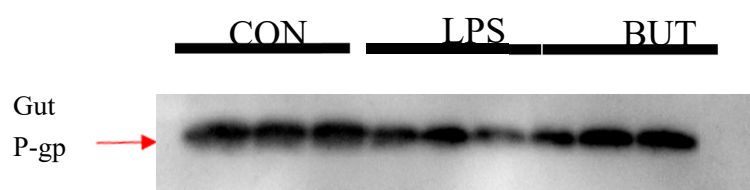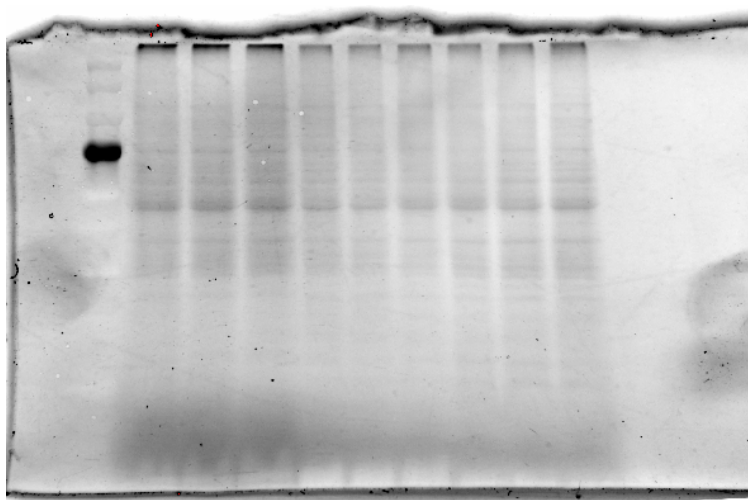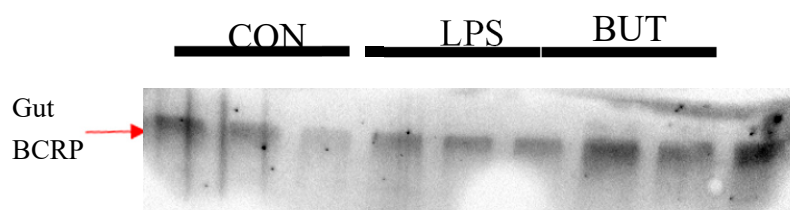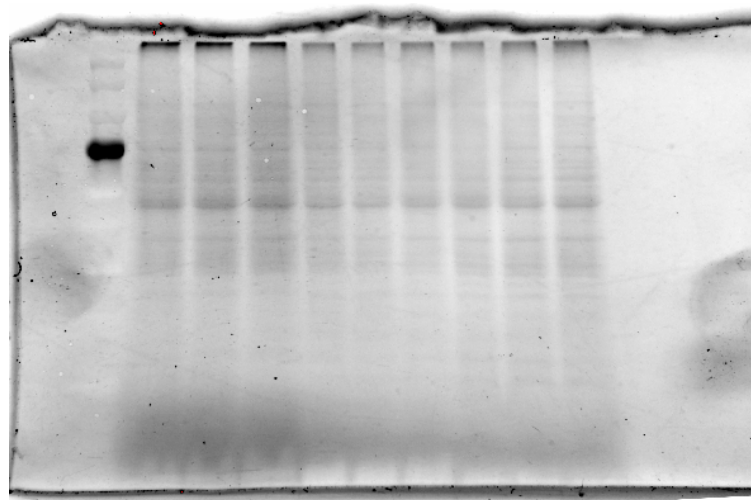

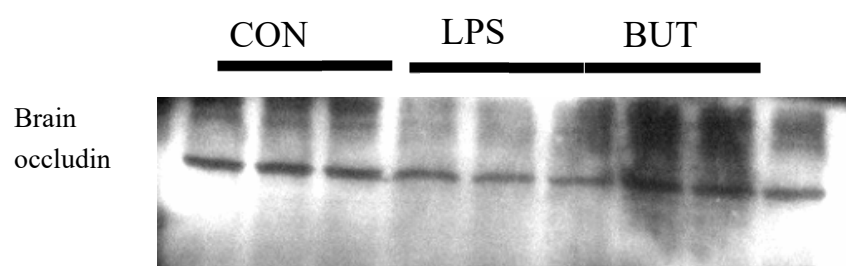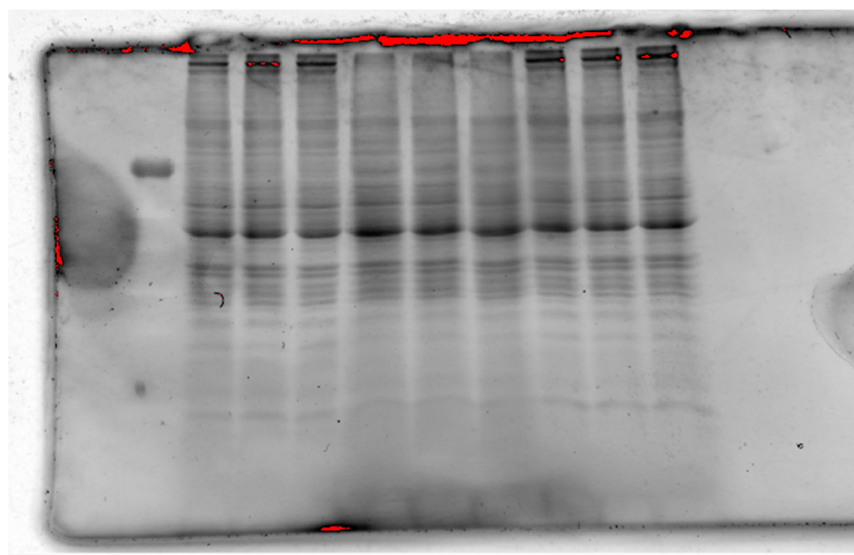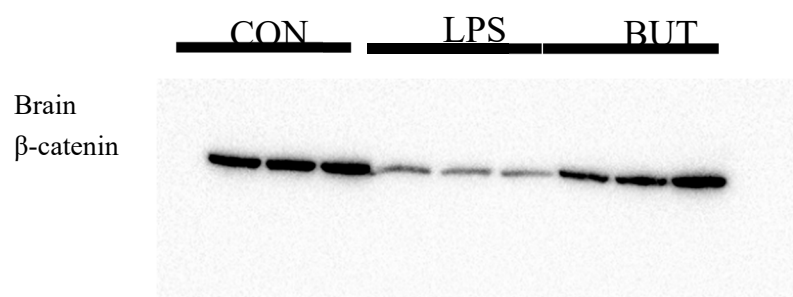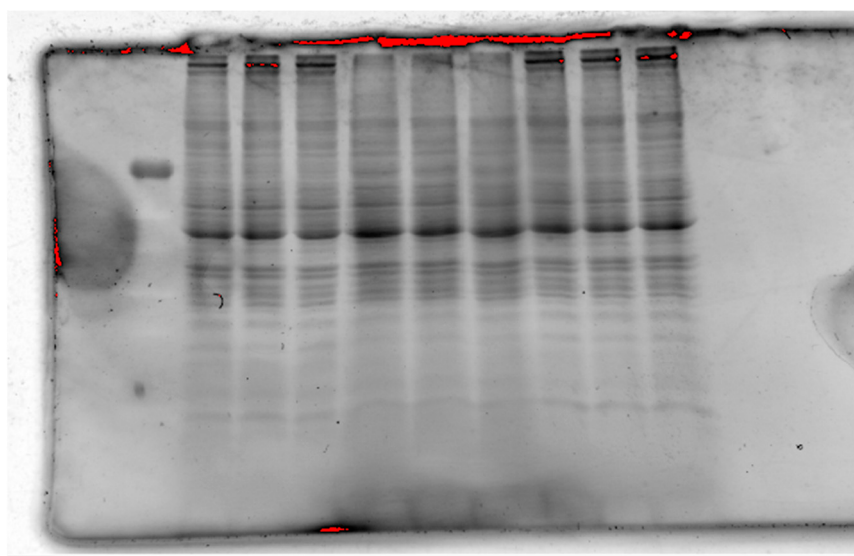

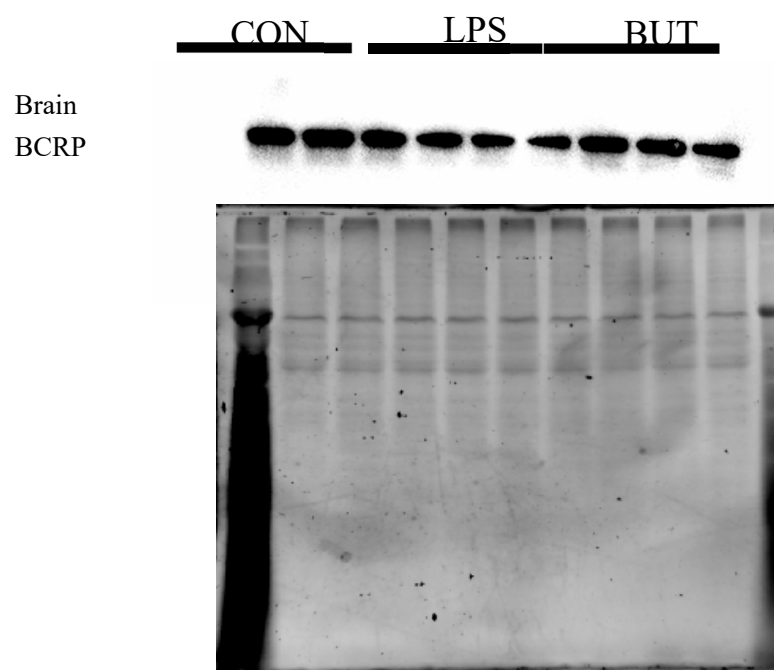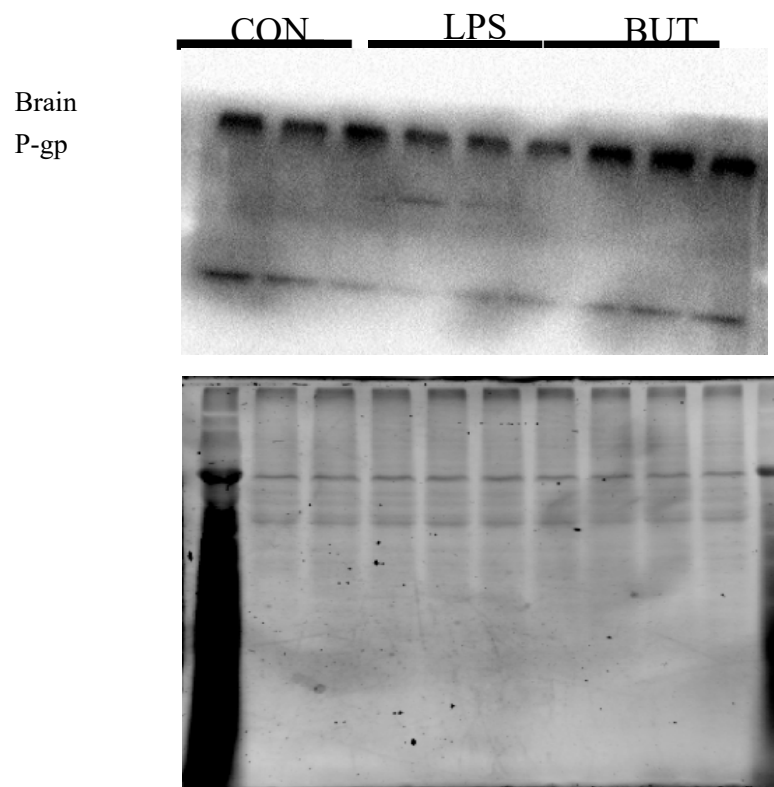

Supplement: Supplementary file 1 [file biosensors-16-00023-s001.zip › Western blot bands and original gels in cell.pdf]
